# Risk of cardiovascular events and death following retinal arterial occlusion and transient monocular visual loss

**DOI:** 10.1038/s41598-026-58854-8

**Published:** 2026-06-19

**Authors:** Josef Huemer, Mario Cortina-Borja, Hagar Khalid, Arvind Chandratheva, Alastair K. Denniston, Jugnoo S. Rahi, Axel Petzold, Pearse A. Keane, Siegfried K. Wagner

**Affiliations:** 1 NIHR Biomedical Research Centre for Ophthalmology, Moorfields Eye Hospital NHS Foundation Trust and UCL Institute of Ophthalmology, London, UK; 2 NIHR Moorfields Biomedical Research Centre, London, UK; 3 Institute of Ophthalmology, University College London, London, UK; 4 Department of Ophthalmology and Optometry, Kepler University Hospital, Linz, Austria; 5 Great Ormond Street Institute of Child Health, University College London, London, UK; 6 Department of Brain Repair and Rehabilitation, Stroke Research Centre, UCL Queen Square Institute of Neurology, London, UK; 7 University of Birmingham, Birmingham, UK; 8 University Hospitals Birmingham NHS Foundation Trust, Birmingham, UK; 9 Great Ormond Street Hospital NHS Foundation Trust, London, UK; 10 Ulverscroft Vision Research Group, University College London, London, UK; 11 Queen Square Institute of Neurology, The National Hospital for Neurology and Neurosurgery (UCLH), University College London, London, UK

**Keywords:** Cardiology, Diseases, Medical research, Risk factors

## Abstract

Acute retinal ischaemic events, ranging from central retinal artery occlusions (CRAO) and branch retinal artery occlusions (BRAO) to transient ischaemic monocular vision loss (TMVL), cause sudden and often severe vision loss, yet their broader implications for systemic vascular health are not well defined. To investigate this, we conducted a large, propensity-matched cohort study including a total of 566 patients at an academic ophthalmic hospital in London, United Kingdom, linking ophthalmic diagnoses to national records of cardiovascular events and mortality. Our findings reveal that patients experiencing a CRAO have a nearly threefold increased risk of a subsequent ischaemic stroke or myocardial infarction, when compared to matched controls. This elevated risk, however, was not apparent in individuals who suffered a BRAO or TMVL, suggesting a distinct risk profile for CRAO. Systemic comorbidities like hypertension and diabetes were prevalent in all groups. Atrial fibrillation was newly diagnosed in 6% of patients with CRAO within one year. Patients with BRAO were more likely to undergo carotid endarterectomy than those with CRAO. The substantial risk of serious cardiovascular morbidity following retinal arterial ischaemia highlights the need to ensure such patients benefit from early treatment and prolonged cardiac monitoring and rigorous implementation of secondary prevention in RAO and suspected embolic TMVL.

## Introduction

Ischaemic events of the retinal arterial vasculature are a cause of acute painless monocular vision field loss. Central retinal artery occlusion (CRAO) and branch retinal artery occlusion (BRAO) lead to irreversible retinal infarction, whereas the ischaemic event in transient monocular vision loss (TMVL), also called amaurosis fugax, is reversible.^1^ The underlying causes and mechanisms of action are comparable to a stroke or transient ischaemic attack (TIA) involving the internal carotid artery territory.^2^ Therefore, CRAO and BRAO are considered part of the stroke spectrum and TMVL that of TIA.^3,4^ The risk of a further stroke after TIA is reported to be 10% in the first week following the event with about half of events occurring within 24 h. ^5,6^ A recent meta-analysis of asymptomatic and symptomatic patients with retinal artery occlusions found contemporaneous acute cerebral ischemia on MRI in 30% of patients with CRAO and 25% of patients with BRAO;^7^ however another MRI-based study assessing patients with CRAO, BRAO and TMVL found that in up to 90% of patients, no additional neurological symptoms were reported, and the brain infarction was labelled as silent.^8^ As shown in two landmark trials, urgent assessment and treatment of patients with TIAs can reduce the risk of further strokes by up to 80%.^9,10^. The list of differential diagnoses for TMVL is long and includes many non-urgent entities, however scenarios such as embolic TMVL require urgent evaluation, underlining the importance of prompt clinical evaluation.^5^.

Here, we report on the incidence of myocardial infarction (MI), ischaemic stroke (IS), carotid endarterectomy (CEA) and death as well as prevalent and incident medical comorbidity burden in patients experiencing CRAO, BRAO and TMVL. We use an individual record linkage approach drawing on ophthalmic diagnoses at a large tertiary eye hospital and hospital admission episodes for the whole of England.

## Results

**Fig. 1:**
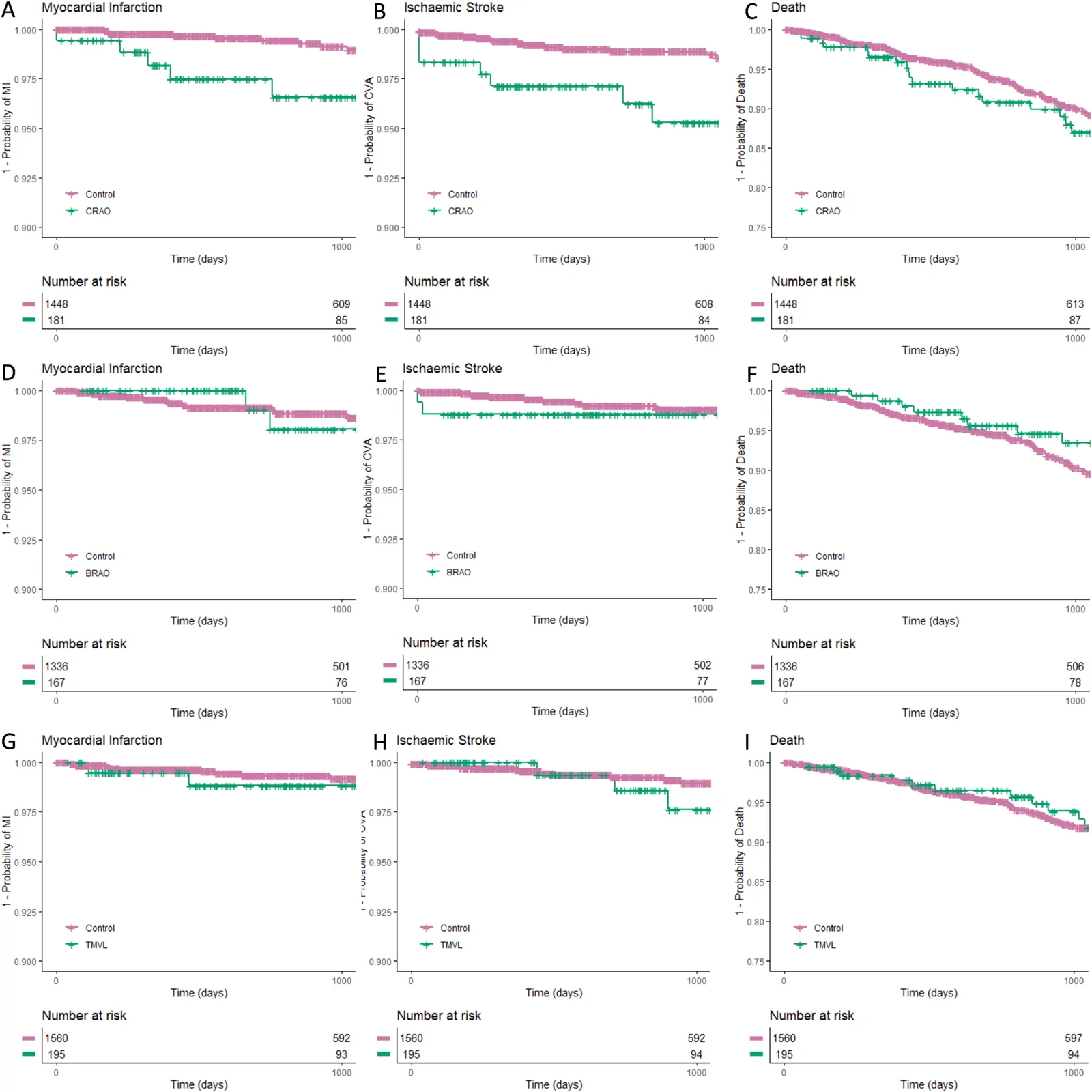
Time-to-event analysis for Myocardial Infarction, Ischemic Stroke and Death. Collage of Kaplan-Meier curves by ophthalmic and systemic event for cases (green) and propensity-matched controls (magenta). (**A**-**C**) depict 1-probability for MI, IS and death respectively for individuals who have experienced a CRAO, (**D**-**F**) for those with a BRAO and (**G**-**I**) for those with TMVL. BRAO: branch retinal artery occlusion, CRAO: central retinal artery occlusion, IS: ischaemic stroke, MI: myocardial infarction, TMVL: transient monocular vision loss.

Between 1st August 2011 and 31st March 2018, 566 patients received a diagnosis of retinal artery occlusion and/or transient monocular visual loss − 190 with CRAO, 178 BRAO and 205 TMVL (i.e. some had more than one diagnosis). Baseline characteristics stratified by each ophthalmic event are shown in Table 1. Good matching between cases and propensity-matched controls was achieved (**Supplementary Table 1**). Time-to-event analysis with Kaplan-Meier curves for MI, IS and death for all three entities are displayed in Fig. 1.

**Table 1:** Baseline sociodemographic data of the cohort stratified by ophthalmic event CRAO = central retinal artery occlusion, BRAO = branch retinal artery occlusion, IS = ischaemic stroke, TMVL = transient monocular visual loss, IQR = interquartile range, SES = socioeconomic status through index of multiple deprivation where 1 is the most deprived and 10 the least deprived decile.

		CRAO n=190	BRAO n= 178	TMVL n= 205
Sex n (%)	Female	68 (35.6)	61 (34.3)	109 (53.2)
	Male	122 (64.4)	117 (65.7)	96 (46.8)
Age median (IQR)	Years	70.1 (61.6.1-78.6)	69.9 (59.0-80.8)	68.1 (58.5-77.7)
Ethnicity n (%)	Asian (South)	22 (11.6)	19 (10.7)	35 (17.1)
	Black	20 (10.5)	13 (7.3)	16 (7.8)
	White	108 (56.8)	109 (61.2)	115 (56.1)
	Other/Unknown	40 (21.1)	37 (20.8)	39 (19.0)
SES median (IQR)	Decile	5 (3-7)	6 (3-8)	5 (1.5-6.5)
Preceding MI	N (%)	5 (2.6)	9 (5.1)	8 (3.9)
Preceding IS	N (%)	4 (2.1)	3 (1.7)	3 (1.4)
Mortality rate	Per 1000 PYAR	29.9 (17.6-47.1)	12.7 (5.0-25.7)	12.5 (5.4-24.2)
Incidence rate - MI	Per 1000 PYAR	9.6 (3.4-20.5)	4.3 (0.7-13.1)	7.2 (2.2-16.8)
Incidence rate -IS	Per 1000 PYAR	13.5 (5.8-26.1)	6.4 (1.6-16.7)	7.1 (2.2-16.6)

### CRAO

A total of 190 patients (64.4% male) with a median age of 70.1 (range 53.1–87.1) years were diagnosed with a CRAO during the study time period (Table 1). During the study period, 34 patients died. Five patients had an incident MI at a median of 322 +/- 175 days including one patient who had an MI one day after CRAO diagnosis. Seven patients had an IS following CRAO with three patients diagnosed within one day after the CRAO diagnosis. Individuals with a CRAO were more likely to experience an MI (hazard ratio [HR]: 2.96, 95% confidence interval [CI]: 1.02, 8.63) and stroke (HR: 2.92, 95% CI: 1.24, 6.90, Table 1) compared to matched controls. One patient had had a CEA prior to their CRAO. Of the five patients, who had a carotid endarterectomy (CEA) following a CRAO, three were within 14 days. Of the 190 patients with a CRAO, 136 had a hospital admission during the study period (Table 3). Individuals frequently had prevalent hypertension (53.7%), diabetes mellitus (18.4%) and hypercholesterolemia (26.5%). Within 1 year, 6% of patients were newly diagnosed with atrial fibrillation (AF) (95% CI: 0.04, 0.09 per PYAR). Five of 136 (3.7%) individuals were diagnosed with giant cell arteritis (GCA) following the event. Exclusion of individuals ultimately diagnosed with GCA neither significantly altered the incidence rate of death, MI, ischaemic stroke (**Supplementary Table 2**) nor the association between CRAO and mortality (adjusted HR 1.46, 95% CI: 0.98, 2.18).

**Table 2:** Association between retinal ischaemic event and incident myocardial infarction, ischaemic stroke and death.

Diagnosis	Myocardial infarction		Ischaemic stroke		Death	
	HR (95% CI)	*p*-value	HR (95% CI)	*p*-value	HR (95% CI)	*p*-value
Central retinal artery occlusion	2.96 (1.02, 8.63)	0.047	2.92 (1.24, 6.90)	0.014	1.48 (1, 2.21)	0.05
Branch retinal artery occlusion	0.80 (0.16, 3.99)	0.79	1.83 (0.55, 6.14)	0.33	0.72 (0.41, 1.28)	0.26
Transient monocular vision loss	1.74 (0.51, 5.88)	0.37	2.07 (0.67, 6.41)	0.21	0.98 (0.62, 1.53)	0.92
Adjusted hazard ratios were estimated through 8:1 propensity-matched Cox proportional hazards models. All models were adjusted for age, sex, hypertension and diabetes mellitus.						
CI: confidence interval, HR: hazard ratio.						

**Table 3:** Medical comorbidities among those with a hospital admission.

Comorbidity	CRAO (*n* = 136)			BRAO (*n* = 96)			TMVL (*n* = 131)		
	Prevalent *n* (%)	Incident *n* (%)	Incidence rate PYAR (95% CI)	Prevalent *n* (%)	Incident *n* (%)	Incidence rate PYAR (95% CI)	Prevalent *n* (%)	Incident *n* (%)	Incidence rate PYAR (95% CI)
Hypertension	73 (53.7)	40 (29.4)	0.44 (0.32, 0.59)	72 (75)	22 (22.9)	0.43 (0.27, 0.63)	83 (63.4)	23 (17.6)	0.24 (0.15, 0.36)
Diabetes Mellitus	25 (18.4)	17 (12.5)	0.06 (0.03, 0.09)	19 (19.8)	5 (5.2)	0.02 (0.01, 0.04)	25 (19.1)	7 (5.3)	0.02 (0.01, 0.05)
Hypercholesterolemia	36 (26.5)	38 (27.9)	0.19 (0.14, 0.26)	37 (38.5)	25 (26.0)	0.17 (0.11, 0.25)	43 (32.8)	21 (16.0)	0.10 (0.06, 0.15)
AF	17 (12.5)	19 (14.0)	0.06 (0.04, 0.09)	15 (15.6)	7 (7.3)	0.03 (0.01, 0.06)	22 (16.8)	5 (3.8)	0.02 (0.01, 0.04)
Asthma	11 (8.1)	6 (4.4)	0.02 (0.01, 0.04)	*	*	0.01 (0.00, 0.03)	13 (9.9)	4 (3.1)	0.01 (0.00, 0.03)
COPD	13 (9.6)	10 (7.4)	0.03 (0.01, 0.05)	7 (7.3)	*	0.03 (0.01, 0.05)	*	*	0.01 (0.00, 0.02)
Angina	20 (14.7)	8 (5.9)	0.02 (0.01, 0.05)	12 (12.5)	11 (11.5)	0.04 (0.02, 0.08)	15 (11.5)	7 (5.3)	0.02 (0.01, 0.04)
CKD	13 (9.6)	18 (13.2)	0.05 (0.03, 0.08)	7 (7.3)	9 (9.4)	0.03 (0.02, 0.06)	10 (7.6)	13 (9.9)	0.04 (0.02, 0.07)
Heart Failure	9 (6.6)	16 (11.8)	0.05 (0.03, 0.07)	8 (8.3)	10 (10.4)	0.04 (0.02, 0.07)	11 (8.4)	7 (5.3)	0.02 (0.01, 0.04)
Hypothyroidism	8 (5.9)	7 (5.1)	0.02 (0.01, 0.04)	5 (5.2)	*	0.01 (0.00, 0.03)	11 (8.4)	5 (3.8)	0.01 (0.01, 0.03)
GCA	0	5 (3.7)	0.01 (0.00, 0.03)	0	0	-	0	*	0.01 (0.00, 0.02)

*Suppressed due to small numbers CI: confidence interval, CKD: chronic kidney disease, COPD: chronic obstructive pulmonary disease, PYAR: person-year at risk. AF: Atrial fibrillation, COPD: Chronic obstructive pulmonary disease, CRAO: central retinal artery occlusion, BRAO: branch retinal artery occlusion, TMVL: transient monocular visual loss, CKD: chronic kidney disease, GCA: Giant cell arteritis, *fewer than 5 cases.

### BRAO

**Fig. 2:**
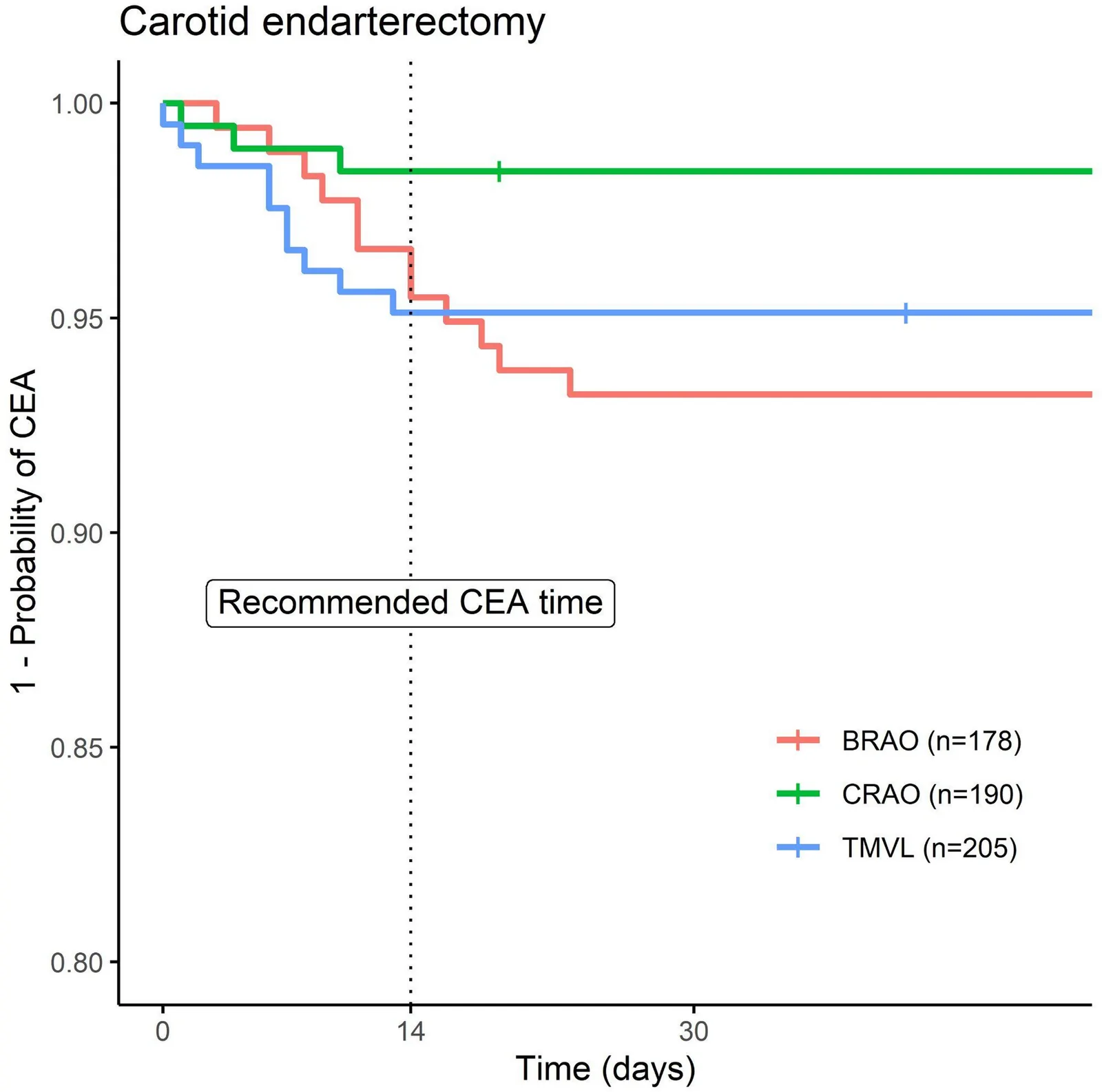
Kaplan-Meier curves for time to carotid endarterectomy among those with central retinal artery occlusion, branch retinal artery occlusion and transient monocular visual loss. The dotted vertical line at 14 days indicates the recommended maximum duration between retinal ischaemic event and undergoing carotid endarterectomy. BRAO: branch retinal artery occlusion, CEA: carotid endarterectomy, CRAO: central retinal artery occlusion, TMVL: transient monocular visual loss.

Of 178 patients who experienced a BRAO during the study period, 117 were male (65.7%) and the median age at presentation was 69.9 (59.0-80.8) years. Only two patients had incident MI following a diagnosis of BRAO with a median time of 707 +/- 43 days. Only two patients had an incident IS (median 18 +/- 553 days), one of which was within 4 weeks following the BRAO. Hazards of MI, IS and death were no different between individuals with a BRAO and propensity-matched controls. Two patients had a previous CEA and 11 patients (6.2%) had one following their BRAO at a median of 12.5 (8.75–16.5) days. Those with a BRAO were significantly more likely to have a subsequent CEA compared to those with CRAO (incidence rate ratio [IRR] 2.84, 95% CI 1.00-8.07, *p* = 0.04, Fig. 2).

### TMVL

Among 205 patients who were diagnosed with TMVL in the study period, the median age was 68.1 (58.5–77.7) years of age and, in contrast to CRAO or BRAO, the majority of patients were female (*n* = 109, 53.2%). Eight individuals had a preceding MI and three had a preceding IS. There were four cases of incident MI (median 702 +/- 733 days) and four cases of incident IS however for neither were the events within 1 year. There was no difference in time to MI, IS or death between those with TMVL and propensity-matched controls. Eleven patients (5.4%) had a CEA following an episode of TMVL at a median of 7 (5-10.75) days (10/12 within 14 days).

## Discussion

In this nested study within the ethnically diverse AlzEye cohort over an approximate 7-year period, we report an elevated risk of MI and IS following CRAO. Among those with CRAO, 6% of patients were newly diagnosed with AF within a year and 5 patients (3.7%) ultimately received a diagnosis of GCA. While we did not observe similarly elevated rates of MI and stroke in those with BRAO and TMVL, approximately 5% of individuals underwent CEA. Collectively, our results highlight the need for urgent evaluation and optimization of cardiovascular risk factors given delayed risk of MI. Long term cardiac monitoring may also be considered to detect AF in those with acute retinal ischaemic events.

Individuals with CRAO had a near 3-fold increased risk of stroke in our study (HR 2.96, 95% CI: 1.02, 8.63) compared to propensity-matched controls. An analysis of US medicare beneficiaries showed in the first and second weeks after CRAO, IS incidence increased 28-fold and 33-fold, respectively, compared to a control group with hip fractures. This is in line with a population based study from South Korea, where the authors discovered a nearly 22-fold increase in IS after three days and a 70-fold rise during the first week following CRAO.^11,12^ This analysis is based on a self-controlled case series, with defined control periods, thus this more extreme estimate is due to the comparator group - our controls were identified through propensity-matching so as to estimate the incremental risk conferred beyond typical medical comorbid factors, such as hypertension and diabetes mellitus. We found an IS incidence rate of 13.5 (5.8, 26.1) per 1000 PYAR following CRAO with 3 patients having a stroke one day after the diagnosis of CRAO, compared to incidence rates of 6.4 (1.6, 16.7) for BRAO and 7.1 (2.2, 16.6) for TMVL. In accordance, the risk for IS appeared highest in the first three to 14 days following the occurrence, according to a study that included all Danish patients with hospital diagnoses of RAO. ^13^ The Danish study also found that the risks for MI and death in patients with RAO were significantly increased after 14 days, with the highest risk occurring between days 14 and 90 (adjusted rate ratios of 1.98 and 1.64, respectively). In our cohort, interestingly we could show an increased risk for developing MI after CRAO to a greater extend and TMVL to a lesser extend after one year, however the risk for BRAO would increase after two years. Whereas RAO are considered to be of embolic origin either caused by carotid artery disease or cardiogenic embolism in the majority of cases and embolic TMVL share the same pathomechanisms, the delayed increased risk for MI in BRAO may be represent the cumulative cardiovascular disease burden in this cohort over time. The pathomechanism of MI is not considered to be embolic but rather a localised disease and the delayed onset in the BRAO cohort could as well represent this increasing cardiovascular risk profile.

The systematic review and meta-analysis by Fallico et al. evaluated that there are no differences of frequency of symptomatic or asymtomatic strokes between patients with CRAO and BRAO.^7^ This is supported by two reviews summarizing that both patients groups are at a highly increased risk of subsequent vascular events and mortality, specifically noting that this elevated risk applies to both CRAO and BRAO patients.^14,15^.

Atrial fibrillation was common across all retinal ischaemia groups in our study with prevalent figures ranging from 12.5% for those with CRAO to 16.8% in those with TMVL. By the end of the study period, over a quarter of individuals affected with CRAO had been diagnosed with the condition. While AF is an established source of cardioembolism and subsequent stroke^16^, its initial paroxysmal nature contributes significantly to the difficulty of establishing the diagnosis, especially at the time of an acute event such as RAO or TMVL. Thus, previously reported investigations of retinal ischemia provide a variable AF prevalence ranging from 4% to 16%.^17–19^ The rate of AF in our cohort was 25% for patients with CRAO, and 20% for both patients with BRAO and TMVL, thus higher than in previously reported studies though we note this pertained to the subgroup of our cohort with hospital admissions. A recent analysis from Zarkali et al. from the Hyperacute Stroke Unit at University College London Hospital, UK found AF in 9% of patients with ischaemic monocular blindness; the authors stated that this is likely an underestimate as only 53% of patients had prolonged cardiac monitoring.^6^ This is supported by a study about patients with CRAO and preexisting implantable cardiac devices, either in situ at the time of the event (27%) or implanted up to one year post index event (73%).^20^ The authors found an incidence of AF in 33.4% of patients at one year after the CRAO event and 49.6% at two years post CRAO, compared with 33.6% and 43.2% in patients with IS and 22.3% and 31.9% at two years in a control group with eight vascular morbidities. Atrial fibrillation should therefore be considered as risk factor of CRAO and indeed, in cases of cryptogenic CRAO, long-term cardiac monitoring over two to three years may be a prudent addition to the diagnostic process. Once the diagnosis of AF is established, optimizing secondary prevention with appropriate anticoagulation with guidance of established risk stratification tools like the CHA2DS2-VASC score, becomes essential.^21^.

Urgent evaluation includes aims to separate embolic from non-embolic TMVL by a thorough assessment of the carotid arteries.^5,22^ Of interest, in our cohorts those with BRAO were significantly more likely to have a CEA compared to those with CRAO. According to the NASCET study, patients with hemodynamically significant carotid stenosis that results in ipsilateral embolic TMVL, cerebral hemisphere TIA, or mild stroke had a 25% 3-year risk of stroke.^22^ In contrast CEA increased the risk of a subsequent stroke from 4.1% to 9.4% in patients with likely non-embolic TMVL.^5^ Timing of surgery for CEA is paramount, as the benefit depends on multiple factors including the degree of the stenosis, or the duration of symptoms. Rothwell et al. analysed pooled data from the European Carotid Surgery Trial and North American Symptomatic Carotid Endarterectomy Trial and demonstrated this variability in patients with 50% or higher stenosis. To prevent one ipsilateral stroke over five years, the number needed to treat (NNT) was highly dependent on timing: NNT was five for those randomised within 2 weeks after their last ischaemic event, versus 125 for patients randomised after more than 12 weeks. Furthermore, the benefit varied by gender (NNT of nine for men versus 36 for women) and age (NNT of five for age 75 years or older versus 18 for younger than 65 years). These critical time windows have subsequently been included in both American Stroke Association and the European Stroke Organisation guidelines.^23–25^ This explains the timely fashion of the surgery, but does not explain why patients in our cohort with CRAO were less likely to undergo the procedure than those with BRAO. While we cannot be certain as to the reason, it may be that patients with CRAO in our study had levels of stenosis exceeding recommended thresholds for CEA, making the benefit surgical intervention less likely. Furthermore, as the age groups and systemic comorbidities in all three groups are comparable, individuals with CRAO may be in overall worse physical state and may not be considered good candidates for high risk vascular surgery as suggested by the much higher mortality rate in those with CRAO (29.9 per 1000 PYAR) vs. those with BRAO (12.7 per 1000 PYAR). Thirdly, this could be influenced by the small number of patients with differential etiologies, such as GCA, contributing to the overall number of patients with CRAO. When previously embolic sources in CRAO and BRAO were compared, no statistically significant subtype difference has been demonstrated regarding carotid plaques versus cardiac sources of embolism.^26^ The observation has been made however, that calcific emboli which are more likely to be of cardiac origin tend to impact proximally without migrating causing CRAO, whereas cholesterol and platelet-fibrin emboli from carotid plaque migrate more readily to distal arteriolar bifurcations therefore leading to BRAO. This may serve as a plausible hypothesis of why BRAO are more associated with CEA than CRAO.^26^.

Given the long term risk of recurrent stroke and MI after CRAO, BRAO and TMVL of ischaemic origin we suggest treating all eligible patients with antiplatelet therapy, statin and antihypertensives if systemic blood pressure is greater than 130/80mmHg.^9,25^ The intensity of initial antiplatelet therapy and statin dose would be guided by the underlying vascular aetiology and those with evidence of associated atherosclerosis on brain and vascular imaging would receive short term dual antiplatelet^26^ with Aspirin and Clopidogrel^27,28^ followed by Clopidogrel monotherapy and Atorvastatin 80mg.^25^.

Our study has some limitations. Firstly, while HES has been considered a sufficient resource for epidemiological research on MI and IS, there will likely be missed cases of such cardiovascular events, so we report minimum population incidence risk and rates^29^. In subsequent work, this risk could be mitigated by linkage to additional resources such as Office for National Statistics mortality data and primary care records. Sudlow et al. observed that HES captures approximately 80% and 65% of MI and IS cases respectively when considering a reference standard of primary care, HES and death certificate records^30^. Mitigating this bias for IS however is that it is standard operating procedure at MEH for all patients presenting with CRAO or BRAO to be discussed with and referred to stroke physicians and for all cases of transient monocular visual loss to be referred accordingly to dedicated rapid access transient ischaemic attack clinics. Secondly, while our study benefits from a case definition of retinal artery occlusion by a trained ophthalmologist with a minimum of three years ophthalmology experience, it is conceivable that there are misdiagnoses, particularly when it comes to embolic versus non-embolic TMVL.^5^ Indeed, it is conceivable that the altering sex distribution within the TMVL group (in contrast to CRAO and BRAO where Males are predominantly affected) may indicate TMVL mimics more frequently seen in female patients, such as migraine. Therefore our hospital has a aforementioned standard operating procedure to separate embolic from non-embolic TMVL and this is also included in the mandatory hospital training. However, it should be noted that ophthalmic diagnostic codes collected in the source EHR used for this report are not for the purposes of financial reimbursement but rather to convey relevant clinical information regarding a patient’s attendance to their primary care physician - thus, one would expect less information bias than typically found when using insurance or other data, which is routinely collected for non-clinical purposes. The risk of mortality after CRAO may be influenced by the 3.7% of patients with GCA in our cohort, potentially inducing a bias, as we have pertained all patients with CRAO in the mortality analysis. Furthermore, there is a theoretical possibility that not all patients with GCA could be excluded. A further limitation concerns the statistical comparison of outcomes across the three ophthalmic cohorts. Direct between-group comparisons of incident myocardial infarction, ischaemic stroke and mortality were performed but did not reach statistical significance, which is most plausibly explained by the limited number of incident events in each subgroup rather than by true equivalence of risk.

In this study, we characterise the cardiovascular outcomes and peri-event multimorbidity in patients experiencing transient and persistent retinal ischaemic events. As well as supporting previous findings of an immediate escalation of stroke incidence following these events, we highlight important cardiological implications in these patients. Firstly, as there is an insidious increase in MI incidence in those with CRAO and secondly, AF represents a condition of high prevalence in this patient group. Taken in conjunction with corroborative evidence on the risk of dysrhythmias following arterial occlusion, these findings support the concept of early treatment and prolonged cardiac surveillance to reduce the substantial burden of cardiovascular disease in patients suffering from retinal ischaemic events. We recommend that, similar to achievements in stroke medicine, secondary prophylaxis should be rigorously implemented in the setting of suspected embolic TMVL.

Methods.

This was a retrospective cohort substudy using data from AlzEye, a longitudinal record-level linkage project which has been previously described in detail.^31^ Relevant to this report, we collected ophthalmic diagnoses of all attendances to the emergency ophthalmology department of adults aged 40 years and over between 1st August 2011 and 1st April 2018 at the principal site of Moorfields Eye Hospital NHS Foundation Trust (MEH). Individual-level ophthalmic records were linked with hospital admissions data using the Hospital Episode Statistics (HES) database overseen by NHS Digital^32^. Self-reported ethnicity was categorised as Asian, Black, White and Mixed/Other as described in previous ophthalmic work in UK Biobank.^33^ Index of multiple deprivation (IMD) decile, the standard area-based measure of deprivation in the UK, was estimated by by obtaining the corresponding rank of the IMD 2015 value from each patient’s postcode through Lower Super Output Areas and aggregating these ranks into deciles.^34^ Those patients who had previously opted out of use of their health data for reasons other than direct care, were excluded as were those with invalid or missing dates of birth, sex or National Health Service (NHS) number, a unique identifier per each individual in the UK. The study received approval from the United Kingdom Health Research Authority (Research Ethics Committee reference: 18/LO/1163, Confidential Advisory Group reference: 18/CAG/0111).

Three forms of retinal ischaemic events were investigated in this report - CRAO, BRAO and TMVL using structured diagnostic codes in the MEH electronic health record system, the Patient Administration System, entered by ophthalmologists with at least 3 years of clinical experience servicing the MEH emergency department. If patients were diagnosed with both BRAO and CRAO, they would be included in both groups for further analysis. The outcomes of MI and IS as well as medical comorbidity data were extracted from Hospital Episode Statistics (HES), a unified record-level national repository of data relating to admitted patient care (APC) for almost all of the English population.^32^ Diagnoses in HES are coded using the International Classification of Diseases 10th revision (ICD).^35^ In line with previous reports, MI was defined as code I21 or I22 ^36–38^, IS as code I63-I64^39^. Carotid endarterectomy (CEA) surgery was coded as L29 through the Operating Procedure Code Supplement (OPCS), a clinical classification system for operative procedures and interventions.^40–42^ Mortality data were derived from the MEH database, which is updated on a two-weekly basis using reports extracted from the NHS National Spine, and is completed on an individual basis by the MEH Data Quality team to ensure accuracy. Mortality data up to the end of the study period, 31st March 2018, was included. Given the source of mortality data from the National Spine and that of cardiovascular events from national hospital admissions data of all NHS hospitals in England, we envisaged minimal loss to follow up (an exception might be if an individual with an NHS number emigrated from the UK during the study period). In addition to looking at all patients with the ophthalmic event under study, we also report on the subgroup of patients who had a hospital admission during the study period.

### Statistical analysis

The data distributions were analysed visually and statistically. Summary statistics were median +/- interquartile range (IQR) for continuous variables and percentages for categorical variables. Categorical variables were compared using the *U*-statistic Permutation test of independence (an iteration of the Pearson’s chi-squared test with exact non-asymptotic Type 1 error control at the nominal level) and continuous variables through the Wilcoxon-Mann-Whitney *U* test^43–45^. Incidence rates were estimated as the number of new events in the study period per person-years at risk (PYAR) with 95% confidence intervals calculated fitting Poisson regression models with the logarithm of the total PYAR as offset term. The at-risk period was defined from the time of ophthalmic event until the earliest of (i) death, (ii) first event (MI or IS) or (iii) conclusion of the data period on March 31st 2018. Those with a previous cardiovascular event were not excluded however prior events are described. We used propensity score matching (PSM) to estimate the average marginal effect of CRAO/BRAO/TMVL on time to event (MI, IS or death)^46^. We matched on age, sex, hypertension and diabetes using a 8:1 greedy nearest neighbour without replacement approach, where PSM was estimated using logistic regression^47^. We assessed balance between controls and cases using standardised mean differences, variance ratios and empirical cumulative density function^48–51^. No exposed units were discarded. There was no missing data for the variables used. Time to event between groups was compared visually with Kaplan-Meier curves. Hazard ratios with cluster-robust standard errors were estimated using Cox proportional hazards models; for survival analysis, individuals with previous CVD events were excluded^52,53^. For comparing the incidence of having a CEA between groups, we estimated the incidence rate ratio (IRR) and tested against the null hypothesis that the IRR was 1. Statistical analyses were conducted in R version 3.6.4 (R Core Team, 2012. R Foundation for Statistical Computing, Vienna, Austria) using the MatchIt ^54^ and survival^55^ packages.

Our report aligns with the Strengthening The Reporting Of Observational Studies in Epidemiology (STROBE) guidelines and its extension, the Reporting of Studies Conducting Using Observational Routinely Collected Health Data (RECORD)^56,57^.

## Data Availability

The data of the AlzEye study are subject to the contractual restrictions of the data sharing agreements between National Health Service England, Moorfields Eye Hospital and University College London and are therefore not available for access beyond the AlzEye research team. National and international collaborations are however welcomed and researchers should contact the Chief Investigator at p.keane@ucl.ac.uk.
